# RBL2/DREAM-mediated repression of the Aurora kinase A/B pathway determines therapy responsiveness and outcome in p53 WT NSCLC

**DOI:** 10.1038/s41598-022-05013-4

**Published:** 2022-01-20

**Authors:** Lei Duan, Ricardo E. Perez, Sarah Calhoun, Carl G. Maki

**Affiliations:** 1 Department of Anatomy and Cell Biology, Rush University Medical Center, 600 S. Paulina Ave, AcFac 507, Chicago, IL 60612 USA; 2 Rush University Medical Center, Chicago, IL 60612 USA; 3 Robert H. Lurie Comprehensive Cancer Center of Northwestern University, Chicago, IL USA

**Keywords:** Cancer, Cell biology, Biomarkers

## Abstract

Wild-type p53 is a stress-responsive transcription factor and potent tumor suppressor. P53 activates or represses genes involved in cell cycle progression or apoptosis in order to arrest the cell cycle or induce cell death. Transcription repression by p53 is indirect and requires repressive members of the RB-family (RB1, RBL1, RBL2) and formation of repressor complexes of RB1-E2F and RBL1/RBL2-DREAM. Many aurora kinase A/B (AURKA/B) pathway genes are repressed in a p53-DREAM-dependent manner. We found heightened expression of RBL2 and reduced expression of AURKA/B pathway genes is associated with improved outcomes in p53 wild-type but not p53 mutant non-small cell lung cancer (NSCLC) patients. Knockdown of *p53*, *RBL2*, or the DREAM component *LIN37* increased AURKA/B pathway gene expression and reduced paclitaxel and radiation toxicity in NSCLC cells. In contrast, pharmacologic inhibition of AURKA/B or knockdown of AURKA/B pathway components increased paclitaxel and IR sensitivity. The results support a model in which p53-RBL2-DREAM-mediated repression of the AURKA/B pathway contributes to tumor suppression, improved tumor therapy responses, and better outcomes in p53 wild-type NSCLCs.

## Introduction

The tumor suppressor protein p53 is a stress-responsive transcription factor and key determinant of cancer therapy responses^1–8^. P53 is activated by chemotherapy, radiation, and other DNA damaging stresses. Activated p53 then initiates transcription programs that arrest the cell cycle or induce apoptosis. P53 is wild-type in ~ 50% of non-small cell lung cancers (NSCLCs)^9^. Not surprisingly, p53 wild-type status has been associated with improved therapy responses and better outcome in NSCLC in several studies^7,9–12^. However, other studies have reported no such association between p53 status and NSCLC outcome^7,13–16^. The reason for these differences is unclear. Factors that determine whether p53 wild-type status associates with improved therapy responses and better outcome in NSCLC (and other cancers) are unknown.

As a transcription factor, p53 can bind the promoter regions of different genes to activate their expression^17–20^. Genes directly activated by p53 are typically involved in cell cycle arrest and apoptosis, such as *CDKN1A* (p21) and *BBC3* (Puma). However, p53 can also repress transcription of an increasingly large number of genes^21–23^. Transcriptional repression by p53 is indirect. Thus, p53 induces expression of p21 that inhibits cyclin-CDK complexes, leading to hypophosphorylation and activation of the pocket proteins RB1, p130, and p107. The hypophosphorylated pocket proteins bind to E2F factors forming RB-E2F and DREAM transcriptional repressor complexes^24–26^. The DREAM complex is composed of DP, p130/p107, E2F4/5, and MuvB. LIN37 is a MuvB protein required for DREAM complex-mediated gene repression^26,27^. The DREAM complex is recruited to cell-cycle genes with E2F and CHR motifs in their promoters and represses their expression^28^. E2F and CHR motifs are typical characteristics of genes with peak expression in S and M phases, respectively. Nearly 300 cell cycle genes have been identified that are repressed through the p53-DREAM pathway^25,26^. Thus, the DREAM complex can function with RB proteins to induce cell cycle arrest and in this way may play important roles in p53-mediated tumor suppression.

Many genes in the Aurora kinase (AURK) pathway are repressed by p53-DREAM^26^. Aurora kinases A and B (AURKA/B) are ser/thr kinases essential for the onset and progression of mitosis^29,30^. AURKA/B phosphorylate G2/M phase substrates to affect different steps in mitosis, including mitotic spindle assembly, centrosome separation, and others. Both AURKA and B have oncogenic activity, and their high expression is associated with worse outcome in different cancers^31–33^. Notably, AURKA/B and p53 regulate each other through a feedback loop in which p53-DREAM represses AURKA/B gene expression, and AURKA/B proteins activate the E3-ligase MDM2 to inhibit p53^34^. Thus, AURKA/B inhibitors may have the dual benefit of inhibiting mitosis while also activating p53^35,36^. AURKA/B inhibitors (e.g. Alisertib) are currently in combination clinical trials in breast cancer, head and neck cancer, advanced lung cancer, and others.

Findings in the current report link the p53-RBL2-DREAM pathway to AURKA/B pathway expression and outcome in p53 wild-type NSCLC patients. Low RBL2 expression and high AURKA/B pathway expression associates with worse outcome in p53 wild-type NSCLCs in the TCGA database. Studies in NSCLC cell lines confirmed that AURKA/B pathway genes are repressed in a RBL2 and p53-dependent way. NSCLC cells with low RBL2 and high AURKA/B expression are largely resistant to NSCLC therapy reagents paclitaxel and radiation, and pharmacologic inhibition of AURKA/B or knockdown of AURKA/B pathway components sensitized cells to these agents. The results demonstrate RBL2-DREAM mediated repression of AURKA/B pathway genes controls therapy responses and outcome in p53 wild-type NSCLCs.

## Results

### Rbl2 expression levels significantly associate with prognosis in WT p53 NSCLC patients

**Figure 1:**
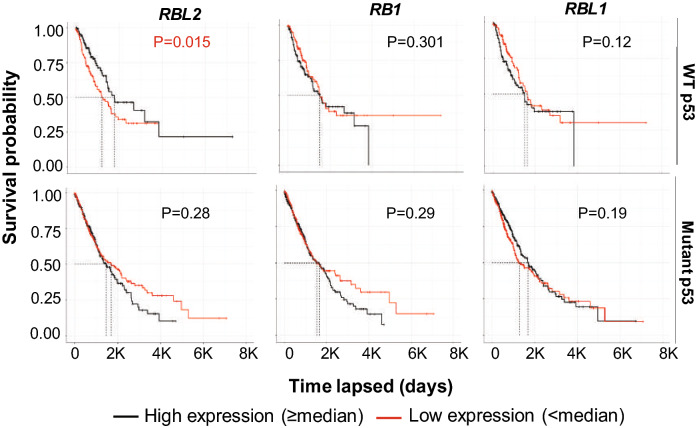
High expression of RBL2 correlates with better prognosis in NSCLC. Expression of DREAM-complex genes are tested for correlation with survival in all 994 NSCLC patients in TCGA. High expression (≥ median) of *RBL2* significantly (p = 0.015) associates with better survival in WT p53 patients but not (p = 0.28) mutant p53 patients. Other DREAM component genes and *RB1/RBL1* do not significantly associate with prognosis (p values indicated).

Gene repression by p53 is indirect and requires one or more members of the Rb-family RB1 (pRb), RBL1 (p107), or RBL2 (p130). We separated NSCLC cases in TCGA into p53 WT and p53 Mutant groups and asked if outcome (survival probability) is associated with expression of each RB family member. We found high RBL2 (p130) expression is significantly associated with better outcome in p53 WT but not mutant cases (Fig. 1). In contrast, we found no association between outcome and expression of RB1 (pRb) or RBL1 (p107), indicating the association with outcome is specific to RBL2.

### RBL2 negatively correlates with DREAM target gene expression in WT p53 NSCLC patients

We speculated gene repression by RBL2 may contribute to the better outcome in p53 WT NSCLCs. Therefore we sought to identify genes that are repressed by RBL2. Gene repression by RBL2 (and RBL1) involves the DREAM transcription repressor complex. Uxa et al.^26^ identified 268 genes that repressed in a DREAM-dependent manner. To identify which of these genes may be repressed by RBL2, we tested for an inverse correlation between RBL2 and all 268 DREAM-regulated genes. 140 genes had an inverse correlation (pearson coefficient < − 0.2) with RBL2 in WT p53 NSCLC patients (Supplementary Tables S1, S2). Notably, some of the genes are also inversely correlated with *RBL2* in mutant p53 NSCLC (Supplementary Table S2). The results suggest that p53 status influences the expression correlation between RBL2 and a substantial subset of DREAM-regulated genes. 22 genes from this 140 gene set are in the AURKA/B pathway (Table 1)^37,38^. The negative correlation between *RBL2* and most of the AURKA/B pathway genes is greatly reduced in mutant p53 tumors (Table 1). This supports the idea that RBL2-mediated repression of the AURKA/B pathway genes is largely WT p53-dependent. Consistent with this, expression levels of these genes are significantly higher in mutant p53 tumors than that in WT p53 tumors (Supplementary Table S3). Interestingly, expression of AURKA gene positively associates with the other AURKA/B pathway genes in WT p53 NSCLC indicated by Pearson coefficient (˃ 0.6). However, this positive correlation is greatly decreased in mutant p53 NSCLC (Supplementary Table S4).

**Table 1:**
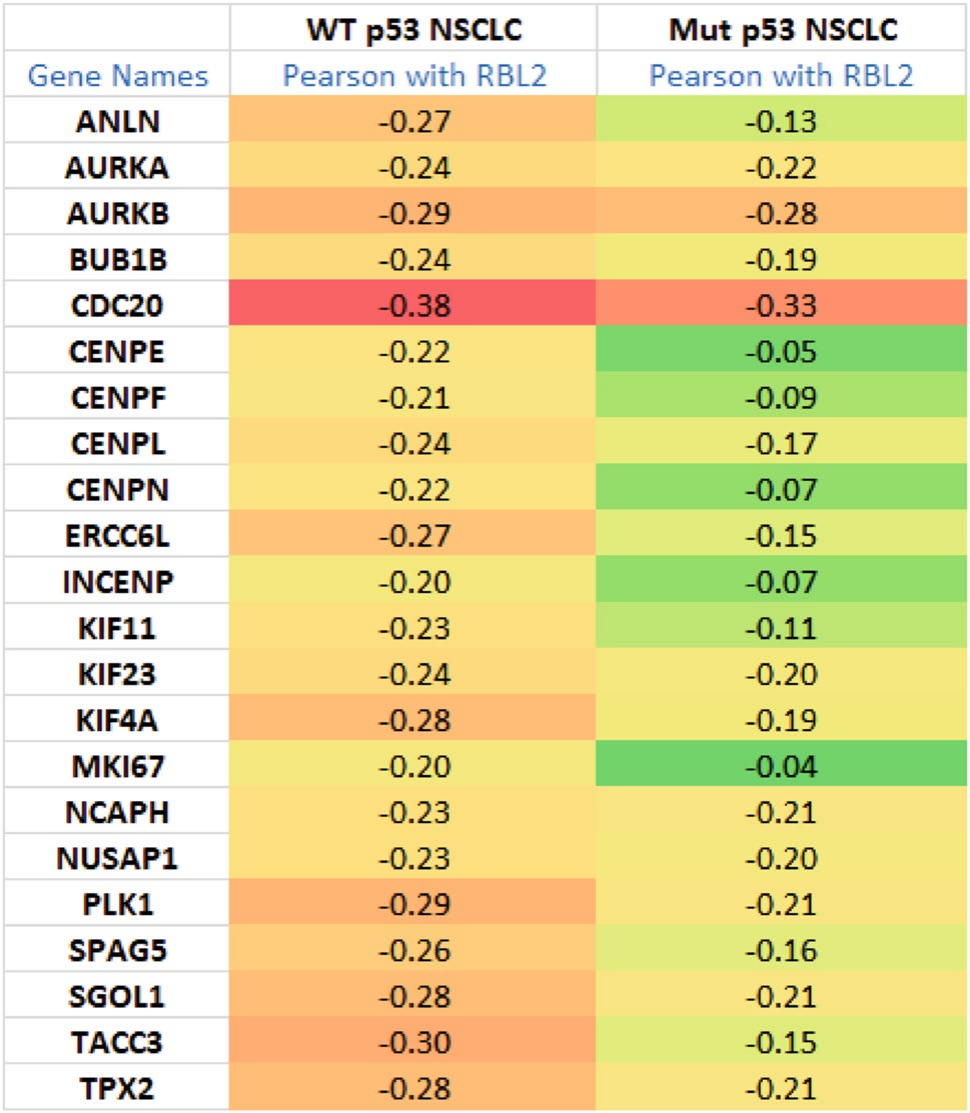
Negative correlation of RBL2 with AURKA/B pathway genes in NSCLC.

**Figure 2:**
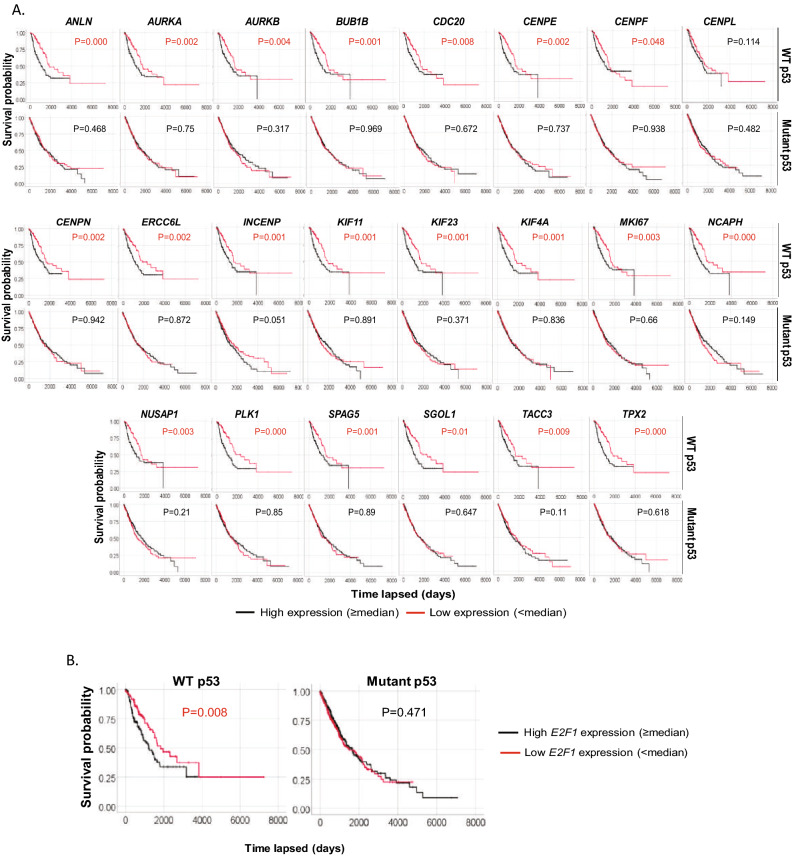
AURKA/B pathway genes associates with prognosis in WT p53 NSCLC patients. (**A**) Expression of the individual AURKA/B pathway genes that negatively correlates with RBL2 in NSCLC patients are tested for correlation with survival in all 994 NSCLC patients. High expression (≥ median) of each gene significantly associates with worse survival in WT p53 but not Mutant p53 patients (p values indicated). (**B**) High expression (≥ median) of *E2F1* significantly associates with worse outcomes in WT p53 but not Mutant p53 patients (p values indicated).

**Figure 3:**
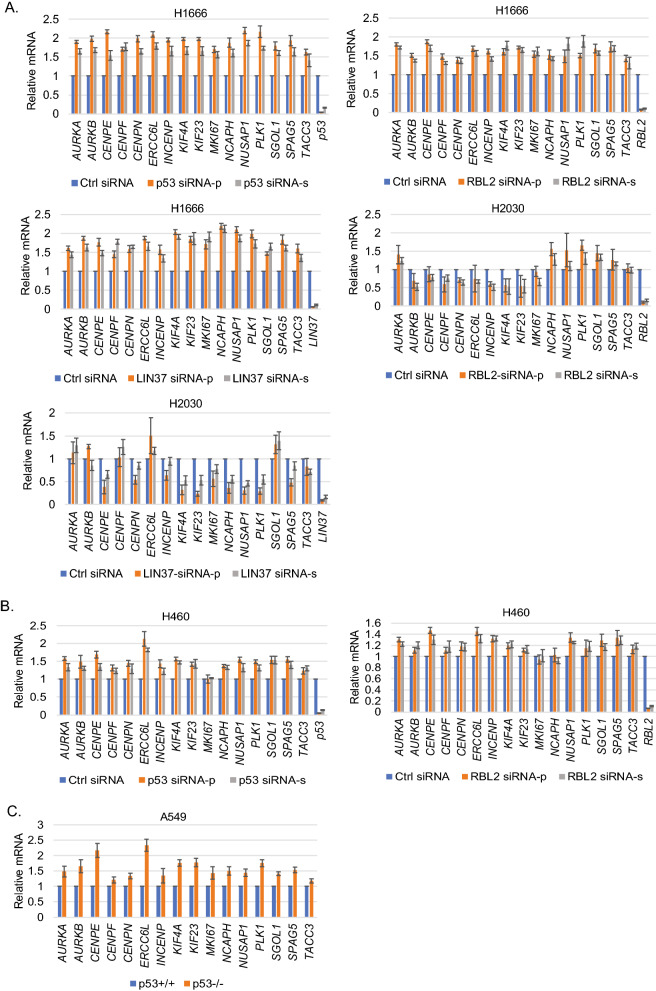
Depletion of p53/RBL2/LIN37 increases expression of the AURKA/B pathway genes. (**A**,**B**) H1666, H2030, H460 were transfected with control siRNA, RBL2 siRNA (pooled and single), p53 siRNA (pooled and single), or LIN37 siRNA (pooled and single) for 24 h and then analyzed for gene expression by qPCR. (**C**) p53+/+ and p53−/− cells were analyzed the indicated gene expression. Average relative mRNA (three independent experiments, one sample each) of the indicated genes are presented with SD indicated. Statistical analyses are shown in Supplementary Table S5–S12.

High expression of cell cycle genes like AURKA/B is well-known to be associated with poor survival across different cancers^39^. We asked if a correlation exists between expression of these AURKA/B pathway genes and survival in WT p53 and mutant p53 NSCLC patients (Fig. 2A). The results showed high expression of each of the genes associates with poor survival in WT p53 patients but not in mutant p53 patients. Later results in Fig. 3 show expression of AURKA/B pathway genes is increased in NSCLC cells upon knockdown of p53, RBL2, or the essential DREAM component LIN37, confirming that these genes are repressed by RBL2-DREAM. These results suggest RBL2-DREAM mediated repression of AURKA/B pathway genes is important for p53-mediated tumor suppression and improved outcome in NSCLC. As expected, expression of p53 activated genes *CDKN1A* (p21), *MDM2*, and *BBC3* (Puma) did not correlate with NSCLC patient outcome in TCGA when the cases were separated by p53 status (Supplementary Fig. S1). The results indicate expression changes of these p53 targets do not by themselves influence NSCLC survival.

Although *RB1* expression does not associate with outcomes in WT p53 NSCLC, we noticed that *E2F1* expression negatively correlated with *RBL2* (Supplementary Table S2) consistent with reports the *E2F1* gene is repressed by RBL2^40^. Kaplan–Meier analysis showed that high expression of E2F1 also associated with significantly reduced survival in WT p53 but not mutant p53 NSCLC patients (Fig. 2B). These results suggest that high expression of RBL2 might contribute to improved outcomes in WT p53 NSCLC patients in part by repressing E2F1 expression.

### p53, RBL2, and LIN37 regulate AURK pathway gene expression and therapy sensitivity in WT p53 NSCLC cell lines

We speculated RBL2-mediated repression of AURKA/B pathway genes could improve outcome in p53 WT NSCLC patients by increasing cancer cell and tumor sensitivity to NSCLC therapy agents. To test this, we first examined RNAseq data from the Cancer Cell Line Encyclopedia (CCLE) to stratify p53 WT NSCLC cell lines based on their relative expression of RBL2. Using median RBL2 expression as a reference, we found H1666 cells express high levels of RBL2 (expression above the median), H460 cells express relatively low RBL2, and HTB53 and A549 cells have RBL2 expression levels at or near the median (Table 2A). We further analyzed the CCLE RNAseq data and found expression of AURKA/B pathway factors is inversely correlated with expression of RBL2. Thus, in H1666 cells that have high RBL2 expression, all 22 AURKA/B pathway genes were expressed below the median while in H460 cells (low RBL2 expression), 18 out of 22 AURKA/B genes were expressed above the median (Table 2A). HTB53 and A549 cells showed intermediate expression of the AURKA/B pathway genes (in HTB53 11 out of 22 AURKA/B genes were expressed above the median, and in A549 14 out of 22 AURKA/B genes were expressed above the median) (Table 2A).

**Table 2:**
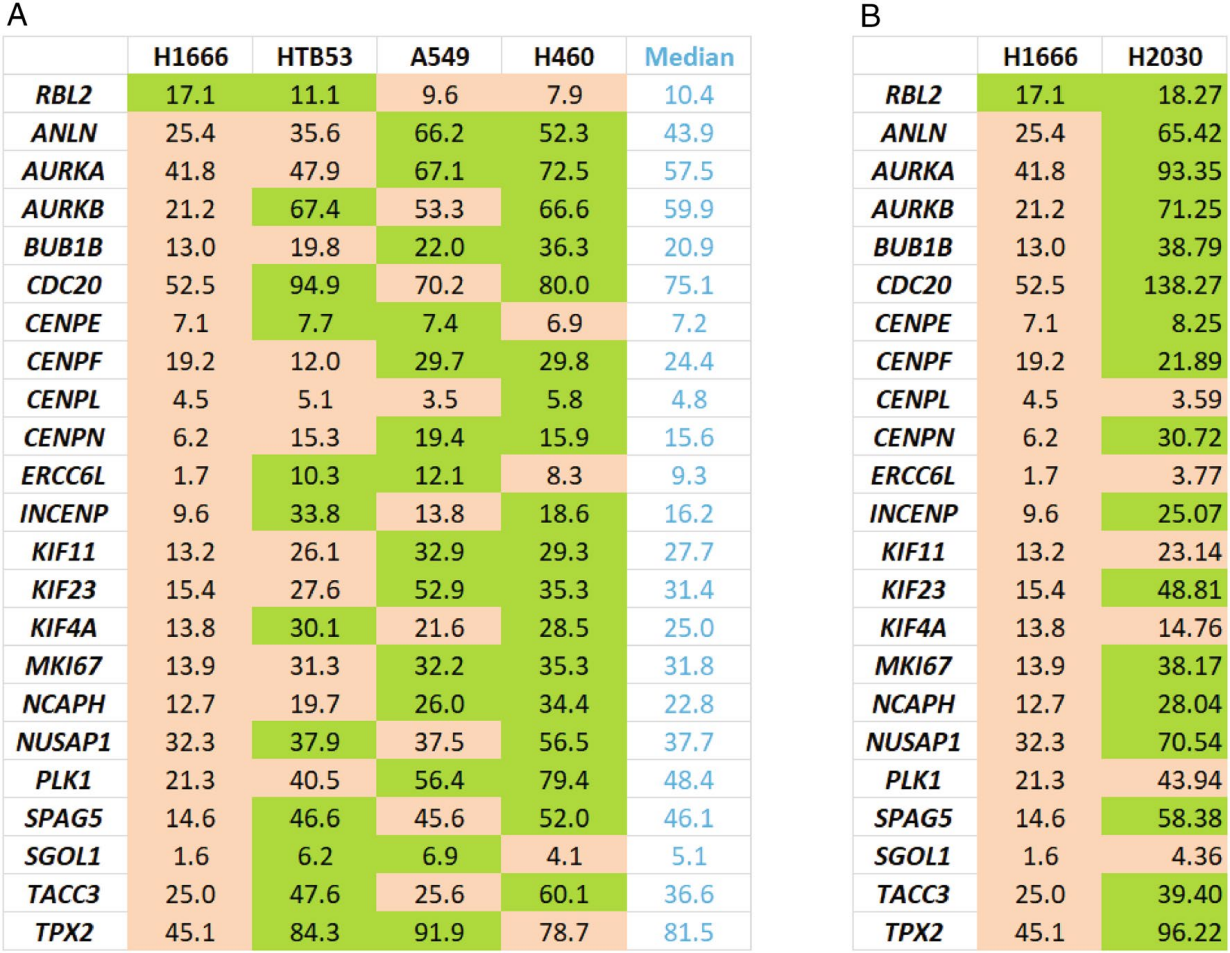
A. WT p53 NSCLC cell lines with different expression levels of RBL2 and AURKA pathway genes. B. RBL2 and AURKA pathway genes in H1666 vs H2030 cell lines.

The findings in Table 2A support a correlation between high RBL2 expression and low expression of AURKA/B genes in p53 WT NSCLC cell lines. To examine this further, we knocked down p53 or RBL2 in H1666 (express high RBL2) and H460 cells (express low RBL2) using two different siRNAs (one pooled siRNA and one single siRNA) and determined expression of a number of the AURKA/B pathway genes. The results showed that knockdown of p53/RBL2 significantly increased expression of the AURKA/B pathway genes in H1666 cells that express high RBL2 (Fig. 3A). However, this effect is relatively mild in H460 cells that express low RBL2 (Fig. 3B). To confirm the AURKA/B pathway genes are repressed by DREAM complex, we further knocked down the DREAM complex component LIN37 that is required for DREAM complex-mediated gene repression^26,27^. Knockdown of LIN37 also significantly increased expression of the AURKA/B pathway genes in H1666 cells (Fig. 3A). Further, we knocked down RBL2 or LIN37 in a mutant p53 NSCLC cell line H2030 that expresses similar level of RBL2 (Table 2B). Unlike H1666 cells, knockdown of RBL2 or LIN37 decreased expression levels of a majority of the AURKA/B pathway genes while modestly increasing expression in 4–5 of the genes (Fig. 3A). Finally, we compared expression of these AURKA/B pathway genes in A549 cells in which p53 is either intact (p53+/+ or Crispr-deleted (p53−/−). A549 p53−/− cells showed higher expression of the AURKA/B pathway genes (Fig. 3C). In total, the results indicate AURKA/B genes are repressed by p53-RBL2 DREAM also in NSCLC cell lines.

**Figure 4:**
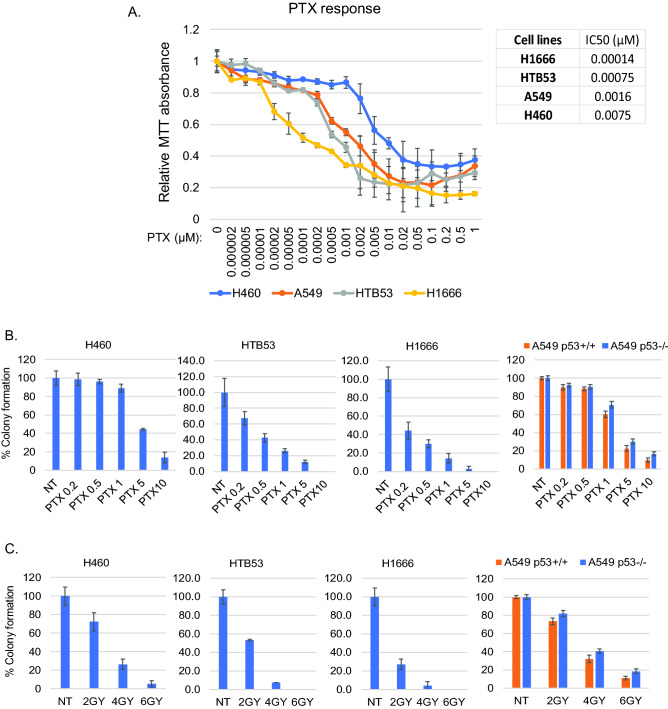
Cells with high RBL2/low AURKA/B pathway genes are sensitive to PTX and irradiation therapies. (**A**) The indicated cell lines were plated in 96-well plates and treated with the indicated doses of PTX for 72 h and then analyzed with MTT. Relative average (8 technical replicate from one experiment) MTT absorbance is presented with SD indicated. (**B**) The indicated cell lines were treated with increasing doses of PTX (μM) for 48 h and then allowed to recover in drug-free media for 2–4 weeks. % average (3 technical replicate from one experiment) formed colonies are presented with SD indicated. There are significant differences (p < 0.05) between H460 and A549 p53+/+ cells treated with 1 μM, 5 μM, and 10 μM of PTX. There are significant differences (p < 0.01) between H460/A549 and HTB53/H1666 cells treated with all doses of PTX. There are significant differences (p < 0.05) between HTB53 and H1666 cells treated with all doses of PTX. There are significant differences (p < 0.05) between A549 p53+/+ and A549 p53−/− cells treated with 1 μM, 5 μM, and 10 μM of PTX. (**C**) The indicated cell lines were treated with 2 Gy, 4 Gy, or 6 Gy irradiation and then allowed to recover for 2–4 weeks. % average (3 technical replicate from one experiment) formed colonies are presented with SD indicated. There is no significant difference (p > 0.05) between H460 and A549 treated with all the doses of irradiation. There are significant differences (p < 0.01) between H460/A549 and HTB53/H1666 cells treated with all doses of irradiation. There are significant differences (p < 0.05) between HTB53 and H1666 cells treated with all doses of irradiation. There are significant differences (p < 0.05) between A549 p53+/+ and A549 p53−/− cells treated with all doses of irradiation.

Next we compared the sensitivity of these NSCLC cell lines to two NSCLC therapy agents, paclitaxel (PTX) and irradiation (IR). First, H1666, HTB53, A549, H460 cells were treated with increasing doses of PTX and cell proliferation and viability analyzed by MTT assay. The results showed that H1666 (high *RBL2* and low AURKA/B pathway gene expression) are most sensitive to PTX while H460 cells (low *RBL2* and high AURKA/B pathway gene expression) are most resistant (Fig. 4A). HTB53 and A549 cells showed an intermediate level of sensitivity. Next, we treated the cells with different doses of PTX and IR and analyzed colony forming ability in H1666, HTB53, H460, A549 p53+/+ and A549 p53−/− cells. H1666 cells were again found to be most sensitive among the cell lines, showing a pronounced reduction in colony formation in response to both PTX and IR (Fig. 4B,C and Supplementary Fig. S2). In contrast, H460 cells were most resistant, while HTB53 and A549 p53+/+ showed intermediate reductions in colony formation in response to both PTX and IR (Fig. 4B,C, Supplementary Fig. S2). Compared to A549 p53+/+ cells, A549 p53−/− cells had significantly increased colonies in response to PTX or IR (Fig. 4B,C). These results suggest high expression of *RBL2* and low expression of AURKA/B pathway genes is associated with PTX and IR sensitivity, while low expression of *RBL2* and high expression of AURKA/B pathway genes is associated with PTX and IR resistance. The PTX and IR sensitivity is in part p53-dependent.

**Figure 5:**
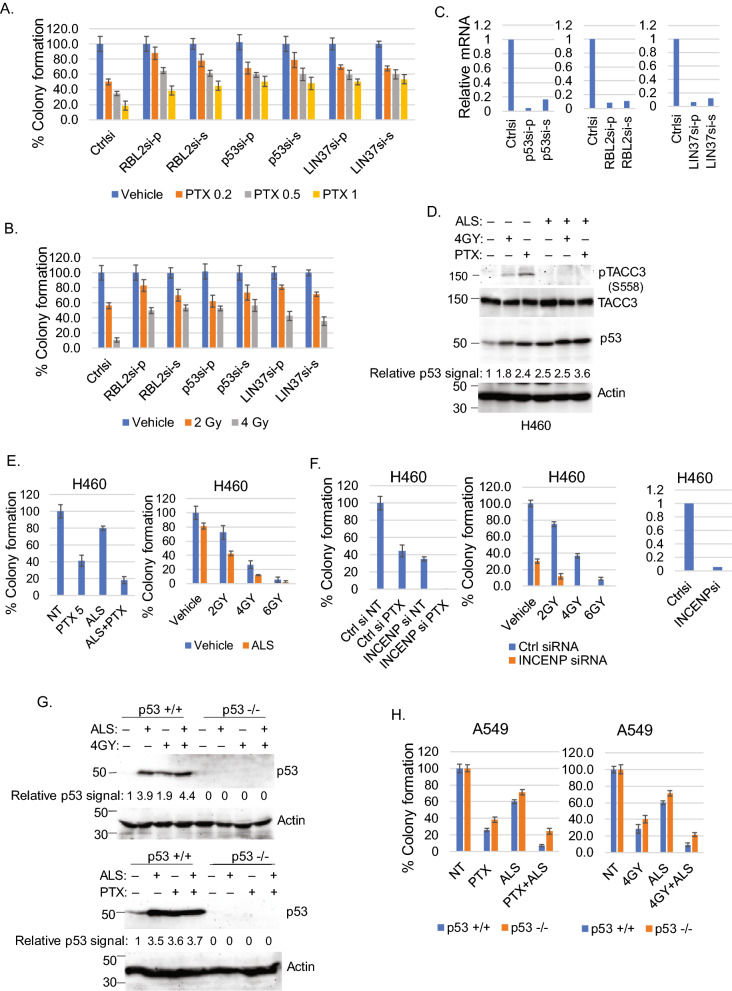
p53/RBL2 regulates response to PTX and irradiation. Alisertib sensitizes cells to PTX/irradiation partially dependent on p53. (**A**,**B**) H1666 and cells were transfected with control siRNA, RBL2 siRNA (pooled and single), p53 siRNA (pooled and single), and LIN37 siRNA (pooled and single), then treated with the indicated doses of PTX (μM) for 48 h or irradiation. Cells were allowed to recover in drug-free media for 2–4 weeks. % average (three technical replicate from one experiment) formed colonies are presented with SD indicated. Statistical analyses are shown in Supplementary Table S13. Reduced gene expression in siRNA transfected cells is shown in (**C**). (**D**) H460 cells were treated with PTX (1 μM) or irradiation (4 Gy) in the absence or presence of alisertib (ALS, 0.1 μM) for 24 h. Lysates were immunoblotted for the indicated proteins (original immunoblot images are shown in Supplementary Fig. S5, representative images of at least two independent experiments). (**E**) and (**F**) H460 cells were treated with PTX (5 μM) or irradiation with or without ALS (50 nM) for 48 h (**E**). H460 cells were transfected with control siRNA or INCENP siRNA and then treated with PTX (5 μM) or irradiation (**F**). Cells were allowed to recover in drug-free media for 2 weeks. % average (three technical replicate from one experiment) formed colonies are presented with SD indicated. Statistical analyses are shown in Supplementary Table S14 and S15. (**G**) p53+/+ and p53−/− A549 cells treated with PTX (1 μM) or irradiation (4 Gy) with or without ALS (0.1 μM) for 24 h. Lysates were immunoblotted for the indicated proteins (original immunoblot images are shown in Supplementary Fig. S5, representative images of at least two independent experiments). (**H**) p53+/+ and p53−/− A549 cells treated with PTX (5 μM) or irradiation (4 Gy) with or without ALS (0.1 μM) for 48 h. % average (three technical replicate from one experiment) formed colonies are presented with SD indicated. Statistical analyses are shown in Supplementary Table S16.

Lastly, we wished to test directly if p53-RBL2-DREAM-mediated repression confers therapy sensitivity in NSCLC cells. To this end, we siRNA-depleted *p53*, *RBL2*, or *LIN37* in H1666 cells, treated the cells with PTX or IR, and monitored colony forming ability. As shown in Fig. 5A–C and Fig. S3, knockdown of *p53*, *RBL2*, or *LIN37* significantly increased colony formation in response to PTX or IR in H1666 cells. This supports the idea that p53-RBL2 DREAM confer PTX and IR sensitivity.

### Aurora kinase inhibition induces p53 and increases therapy sensitivity in p53 WT NSCLC cells

AURKA and AURKB have been reported to phosphorylate and activate MDM2^41,42^, leading to p53 degradation. We reasoned that if high expression of AURKA/B contribute to therapy resistance in p53 WT NSCLC cells, then inhibition of AURKA/B would sensitize the cells to therapy and this would occur in a manner that is at least partially p53-dependent. To test this, we treated the relatively insensitive H460 cells with either vehicle or the AURKA/B inhibitor alisertib in combination with PTX or IR. The microtubule stability regulator TACC protein is a substrate of AURKA^43^. We monitored phosphorylation of TACC3 to confirm Alisertib was active. As shown in Fig. 5D, TACC phosphorylated at S558 (AURKA phosphorylation site) was increased in H460 cells treated with PTX and IR but not in cells co-treated with Alisertib, confirming Alisertib inhibited AURKA. Importantly, Alisertib significantly sensitized H460 cells to both PTX and IR (Fig. 5E and Supplementary Fig. S4A–D), consistent with the idea that high AURKA/B promote therapy resistance in these cells. To examine this further, we tested if knockdown of an AURKA/B pathway factor would increase PTX/IR sensitivity. INCENP (Inner Centromere Protein) plays an important role in the aurora kinase pathway by activating AURKB. As shown in Fig. 5F and Supplementary Fig. S4E–H, *INCENP* knockdown also significantly reduced colony formation in H460 cells basally and in combination with PTX or IR, further supporting that the AURKA/B pathway contributes to therapy resistance. Notably, p53 levels were modestly increased in PTX/IR-treated A549 cells, but increased to a higher level in cells treated with Alisertib alone or Alisertib plus PTX/IR (Fig. 5G). The increase in p53 by Alisertib treatment is consistent with AURKA/B maintaining p53 at low levels. Lastly, we found that alisertib stabilized p53 in A549 p53+/+ cells (Fig. 5G) and sensitized these cells to PTX and IR to a slightly greater extent than A549 p53−/− cells (Fig. 5H and Supplementary Fig. S6). This is consistent with the idea that p53 contributes to therapy-sensitization by Alisertib. In total, the results support p53-RBL2-mediated repression of AURK pathway genes contributes to improved therapy responses and better patient outcome in p53 WT NSCLCs.

## Discussion

P53 functions as a tumor suppressor by activating or repressing genes involved in cell cycle progression and apoptosis. Wild-type p53 is activated in response to chemotherapeutic drug treatment, radiation, and other stresses. Accordingly, p53 wild-type status has been associated in several studies with improved tumor therapy responses and better patient outcomes. However, other studies have reported no such association. The reason for these differences is unclear, but could result from reduced expression of p53 effector molecules in p53 wild-type cancers or heightened expression of p53 negative regulators, such as MDM2. The ability of p53 to repress transcription requires repressive members of the RB-family (RBL1, RBL2) and formation of a transcription repressor complex termed DREAM. However, the role of RBL1/2 and DREAM in p53-mediated tumor therapy responses and/or patient outcomes has not been established. Results from the current study indicate transcription repression via RBL2-DREAM contributes to tumor therapy responses and improved patient outcomes in p53 WT NSCLC.

High RBL2 expression was associated with better outcome in p53 wild-type but not p53 mutant NSCLCs. This supports the idea that RBL2 contributes to p53-mediated tumor suppression in this cancer. Uxa et al. identified 268 genes that are repressed through the p53-DREAM pathway. From these we identified 140 genes whose expression is inversely correlated with *RBL2.* The inverse correlation with *RBL2* suggests these genes may be repressed in a RBL2-DREAM-dependent way. Notably, however, although the inverse correlation of these 140 genes with RBL2 is stronger in WT p53 tumors than mutant tumors, 82 of the genes remain negatively correlated with *RBL2* in mutant p53 tumors (Supplementary Table S2). This implies that RBL2 can also repress transcription of genes in a manner independent of WT p53. Indeed, in G0 and early G1 phase, the CDK inhibitor p27 can bind and inactivate cyclin E-CDK2 complexes and block CDK2-mediated phosphorylation of p107/p130 which is p53-independent^44^. Interestingly, Kim et al. recently showed that DREAM complex can repress a subset of genes in a p53-independent manner, indicating other factors such as PAF can additionally regulate DREAM activity to repress cell cycle genes^45^. Future studies may reveal more factors and mechanisms underlying p53-independent DREAM complex activities. It is noteworthy that in the TCGA dataset RBL2 mRNA levels have a significantly negative correlation with AURKA pathway gene levels in WT p53 cases. However, in a fraction of cases both RBL2 and AURKB pathway gene levels are high, suggesting the ability of RBL2 to repress transcription is not purely determined by gene expression levels. Other regulatory factors that control the phosphorylation of RBL2 and/or its localization may affect the ability of RBL2 to repress transcription.

22 of the genes we identified are involved in the AURKA/B pathway that regulates mitosis. Some of these genes have been found in previous studies to be associated with prognosis in NSCLC patients^46–51^, though their relationship with wild-type p53 has not been indicated. Importantly, we found expression of each of these 22 genes associates with outcome only in wild-type p53 NSCLC patients but not mutant p53 patients. Thus, reduced expression of these 22 genes is significantly associated with improved outcome in p53 wild-type but not mutant NSCLCs, while heightened expression of these genes associates with worse outcome in p53 wild-type but not mutant NSCLC patients. The results suggest p53 and RBL2-mediated repression of these AURKA/B pathway genes may contribute to tumor suppression and improved outcome in patients with p53 wild-type NSCLC tumors. How might heightened expression of AURKA/B and pathway genes lead to worse outcome in p53 wild-type NSCLCs? Both AURKA and AURKB have been reported to phosphorylate p53 and/or MDM2, leading to inhibition of p53 through degradation. Consistent with that, our results showed that Alisertib stabilized p53. This stabilization likely resulted from either a block in AURKA/B-mediated phosphorylation and activation of MDM2, or from mitotic stress caused by Alisertib that could stabilize p53. Thus, it is reasonable to believe that high expression of AURKA/B may suppress p53 function to promote worse prognosis in the wild-type p53 patients. Presumably, high expression of other AURKA/B pathway genes contribute to worse outcome by stimulating mitosis and overall aggressiveness. However, if high expression of AURK pathway genes contributes to worse outcome by increasing mitosis and aggressiveness, then one might expect this would be true regardless of p53 status, which was not the case (Fig. 2). We speculate this is due to the coordinated regulation of these genes that seems to occur in p53 wild-type tumors. Specifically, we found that all 22 genes are coordinately high or low in p53 wild-type NSCLCs indicated by positive correlation between AURKA and the other AURKA/B pathway genes (Supplementary Table S4), while in p53 mutant cases the genes are randomly expressed at high or low levels and in a non-coordinated way. One possibility is that the AURKA/B pathway genes and the proteins they encode need to be coordinately upregulated in order to efficiently increase mitosis and aggressiveness to impact patient outcome.

Some of the AUKRA/B pathway genes in our 22 member gene set have previously been implicated in promoting resistance to chemotherapies^32,52–55^. We compared p53 wild-type NSCLC cell lines that express high RBL2 and low AURKA/B pathway genes with cell lines that express low RBL2 and high AURKA/B pathway genes for their sensitivity to PTX and IR. Cells with high RBL2 and low AURKA/B pathway expression showed increased PTX and IR sensitivity. Furthermore, knockdown of *p53 RBL2*, or *LIN37* increased the AURKA/B pathway gene expression and conferred PTX and IR resistance to these cells. By contrast, pharmacologic inhibition of AURKA/B or knockdown of INCENP (AURKA/B pathway factor) increased PTX and IR sensitivity in cells with low RBL2 and high AURKA/B pathway expression (H460 cells). These results indicate that p53-RBL2-DREAM mediated gene repression of the AURKA/B pathway contribute to NSCLC therapy response (PTX, IR). The AURKA/B inhibitor Alisertib increased p53 levels and, consistent with others findings^35,36^, we found alisertib sensitized NSCLC cells to PTX and IR in a manner that was at least partly p53-dependent. Finally, it is worth noting that besides the AURKA/B pathways genes, the remaining 118 genes that are repressed by the p53-RBL2-DREAM pathway may also be important for therapy responsiveness and outcome in NSCLC.

In summary, our study has found RBL2-DREAM mediated repression of AURKA/B pathway genes associates with improved therapy responses and better outcomes in p53 WT NSCLC. Future studies using large cohorts of clinical patients may establish RBL2 and AURKA/B as predictive markers for therapy sensitivity and prognosis in WT p53 NSCLC patients.

## Methods

### Cell lines and reagents

NSCLC cell lines H1666, HTB53, A549, and H460 are acquired from ATCC Crispr p53 KO A549 (p53−/−) cells and control parental A549 (p53+/+) cells are a generous gift from Dr. William Hahn^56^ (Dana-Farber Cancer Institute). Cells were grown in DMEM medium, with 10% fetal bovine serum (FBS), penicillin (100 U/mL) and streptomycin (100 µg/mL). Alisertib and paclitaxel were obtained from Selleck Chemicals.

### TCGA and CCLE RNAseq databases and bioinformatics analysis

P53 mutation status was obtained from the Pan-lung TCGA^57^ in cbioportal (www.Cbioportal.org). NSCLC Gene expression and survival data were downloaded from proteinatlas (proteinatlas.org/pathology/lung + cancer). All 994 patients from this dataset were tested for association of p53 status or gene expression levels with survival by Kaplan–Meier curve (IBM SPSS). Patients with ≥ median gene expression levels (FKPM) are taken as high expression (~ 50% of patients) and < median are taken as low expression. Gene expression correlation is analyzed with Pearson co-efficiency using Microsoft Excel.

CCLE RNAseq gene expression (RPKM) database^58^ for 1019 cell lines (CCLE RNAseq genes rpkm 20180929.gct.gz) was downloaded from https://portals.broadinstitute.org/ccle/data under Current Data. The RBL2/AURKA/B pathway gene expression was extracted from the database.

All methods were carried out in compliance with relevant guidelines and regulations.

### Immunoblotting

Whole cell extracts were prepared by scraping cells in lysis buffer (150 mM NaCl, 5 mM EDTA, 0.5% NP40, 50 mM Tris, pH 7.5), resolved by sodium dodecyl sulfate polyacrylamide gel electrophoresis (SDS-PAGE) and transferred to polyvinylidene difluoride membranes (Thermo Fisher Scientific). Antibodies to p53 (DO-1, SC-126) and β-actin (C4, SC-47778) were from Santa Cruz. Antibodies phospho-TACC3 (S558, #8842) and TACC3 (#8069) were from Cell Signaling. Primary antibodies were detected with goat anti-mouse or goat anti-rabbit secondary antibodies conjugated to horseradish peroxidase (Life Technologies), using Clarity chemiluminescence (BIO-RAD).

### siRNA-mediated transient knockdown

Pooled p53 siRNA, RBL2 siRNA, LIN37 siRNA and INCENP siRNA (On-target plus smart pool) and Control siRNA (On-target plus siControl non-targeting pool) were purchased from Dharmacon. Single siRNA for p53 (GAGGUUGGCUCUGACUGUA), RBL2 (CACUAACUGGUGUUAGGUA), LIN37 (CUCAGACACCCACAAUAAG) were obtained from Sigma-Aldrich. Cells were transfected according to the manufacturer's guidelines using DharmaFECT I reagent.

### RNA isolation and real-time quantitative PCR analysis

Total RNA was prepared using Total RNA Mini Kit (IBI Scientific, IA); the first cDNA strand was synthesized using High Capacity cDNA Reverse Transcription Kit (Applied Biosystems, CA). Manufacturers’ protocols were followed in each case. The PCR primers for the indicated genes are listed in Supplementary Table S5. SYBR green PCR kit (Applied Biosystems) was used according to the manufacturer’s instructions. AB7300 system was used as follows: activation at 95 °C; 2 min, 40 cycles of denaturation at 95 °C; 15 s and annealing/extension at 60 °C; 60 s, followed by melt analysis ramping from 60 to 95 °C. Relative gene expression was determined by the ΔΔC_t_ method using β-Actin to normalize. PCR reaction was conducted in technical triplicate and average CT values were used to calculate relative expression of genes.

### Exposure of cells to irradiation

Cells were exposed to X-ray irradiation at room temperature in a RS-2000 X-ray Biological System (Rad Source Technologies, Buford, GA) at a dose rate of 2.64 Gy/min. Cells were treated with the following doses: 2, 4, 6 Gy.

### Colony formation assay

Cells were plated in 6-well plates with 200 cells/well in triplicate for 24 h. Cells were then treated with paclitaxel for 24 h or irradiation once and then released of drugs. Cell were allowed to recover for 2–5 weeks to form colonies. Colonies were stained with 1% methylene blue (Sigma) in ethanol and number of positive colonies was counted. Experiments are conducted in triplicate and repeated at least one more time. Average value from one representative experiment is presented with SD indicated as error bars. For siRNA transfected cells, cells were detached 24 h after transfection and then plated accordingly for different treatments.

### Statistical analysis

One-way analysis of variance (ANOVA) and Student's *t*-test were used to determine the statistical significance of differences among experimental groups. Student's *t*-test was used to determine the statistical significance between control and experimental groups. For Kaplan–Meier survival analysis, Log-Rank test was used to determine significance between patient groups with high or low expression of the genes.

## Supplementary Information

Supplementary Information.
