# Author Correction: RBL2/DREAM-mediated repression of the Aurora kinase A/B pathway determines therapy responsiveness and outcome in p53 WT NSCLC

**DOI:** 10.1038/s41598-022-08722-y

**Published:** 2022-03-16

**Authors:** Lei Duan, Ricardo E. Perez, Sarah Calhoun, Carl G. Maki

**Affiliations:** 1 Department of Anatomy and Cell Biology, Rush University Medical Center, 600 S. Paulina Ave, AcFac 507, Chicago, IL 60612 USA; 2 Rush University Medical Center, Chicago, IL 60612 USA; 3 Robert H. Lurie Comprehensive Cancer Center of Northwestern University, Chicago, IL USA

Correction to: *Scientific Reports* 10.1038/s41598-022-05013-4, published online 20 January 2022

The original version of this Article contained an error.

In the second paragraph of the Introduction,

“Thus, p53 induces expression of p21 that inhibits cyclin-CDK complexes, leading to hypophosphorylation and inactivation of the pocket proteins RB1, p130, and p107.”

now reads:

“Thus, p53 induces expression of p21 that inhibits cyclin-CDK complexes, leading to hypophosphorylation and activation of the pocket proteins RB1, p130, and p107.”

The original Article has been corrected.

