# Supplementary material for: RBL2/DREAM-mediated repression of the Aurora kinase A/B pathway determines therapy responsiveness and outcome in p53 WT NSCLC
Source: Sci Rep. 2022 Jan 20;12:1049. doi: 10.1038/s41598-022-05013-4 (PMC8776870; doi:10.1038/s41598-022-05013-4)
Supplement: Supplementary file 1 — Supplementary Information. [file 41598_2022_5013_MOESM1_ESM.pdf]

Supplemental data

Figure legend

Figure S1. Figure 1. p53 direct target genes do not correlate with prognosis in NSCLC. 994 patients were tested for association of p53 status or gene expression levels with survival by Kaplan-Meier curve. Patients with ≥median gene expression levels are taken as high expression (~50% of patients) and <median are taken as low expression. High expression of *MDM2*, *CDKN1A*, or *BBC3* does not significantly (p values shown) associate with survival of the patients.

Figure S2. A. Three individual replicate of the graphs in Fig. 5B. B. Three individual replicate of the graphs in Fig. 5C. C. Representative images of colonies for the graphs in Fig. 5B. D. Representative images of colonies for the graphs in Fig. 5C.

Figure S3. A. Three individual replicate of the graphs in Fig. 6A. B. Three individual replicate of the graphs in Fig. 6B. C. Representative images of colonies for the graphs in Fig. 6A. D. Representative images of colonies for the graphs in Fig. 6B.

Figure S4. A and C. Three individual replicate of the graphs in Fig. 6E. B and D. Representative images of colonies for the graphs in Fig. 6E. E and G. Three individual replicate of the graphs in Fig. 6F. F and H. Representative images of colonies for the graphs in Fig. 6F.

Figure S5. Immunoblot images in Fig 6B and 6E.

Figure S6. A and C. Three individual replicate of the graphs in Fig. 6H. B and D. Representative images of colonies for the graphs in Fig. 6H.

Figure S1

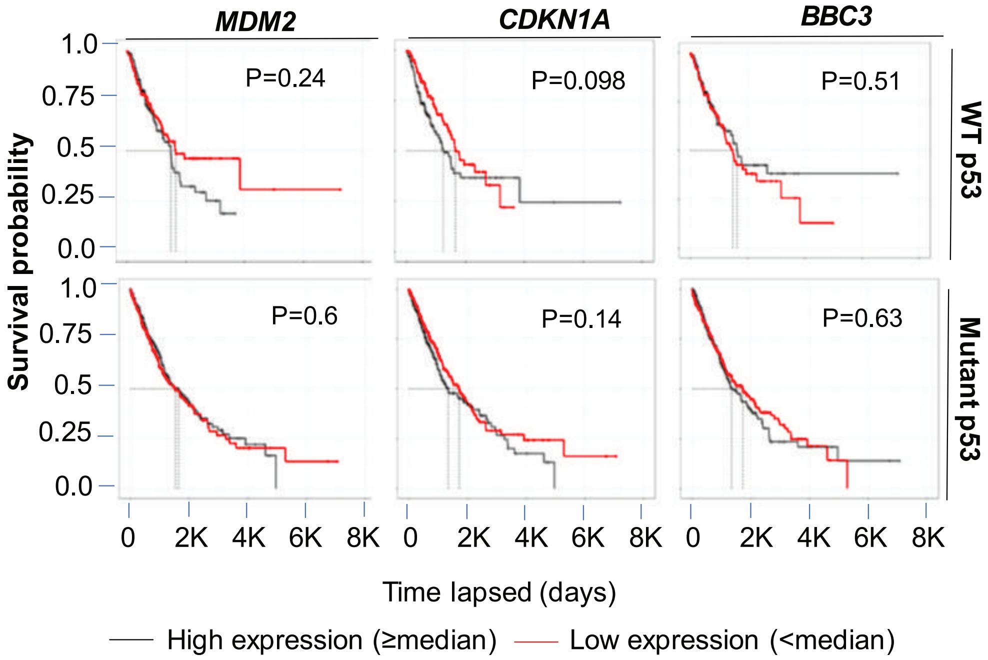

MDM2
CDKN1A
BBC3
P=0.24
P=0.098
P=0.51
P=0.6
P=0.14
P=0.63
WT p53
Mutant p53
Survival probability
1.0
0.75
0.5
0.25
0.0
0
2K
4K
6K
8K
Time lapsed (days)
High expression (≥median)
Low expression (<median)

Figure S2

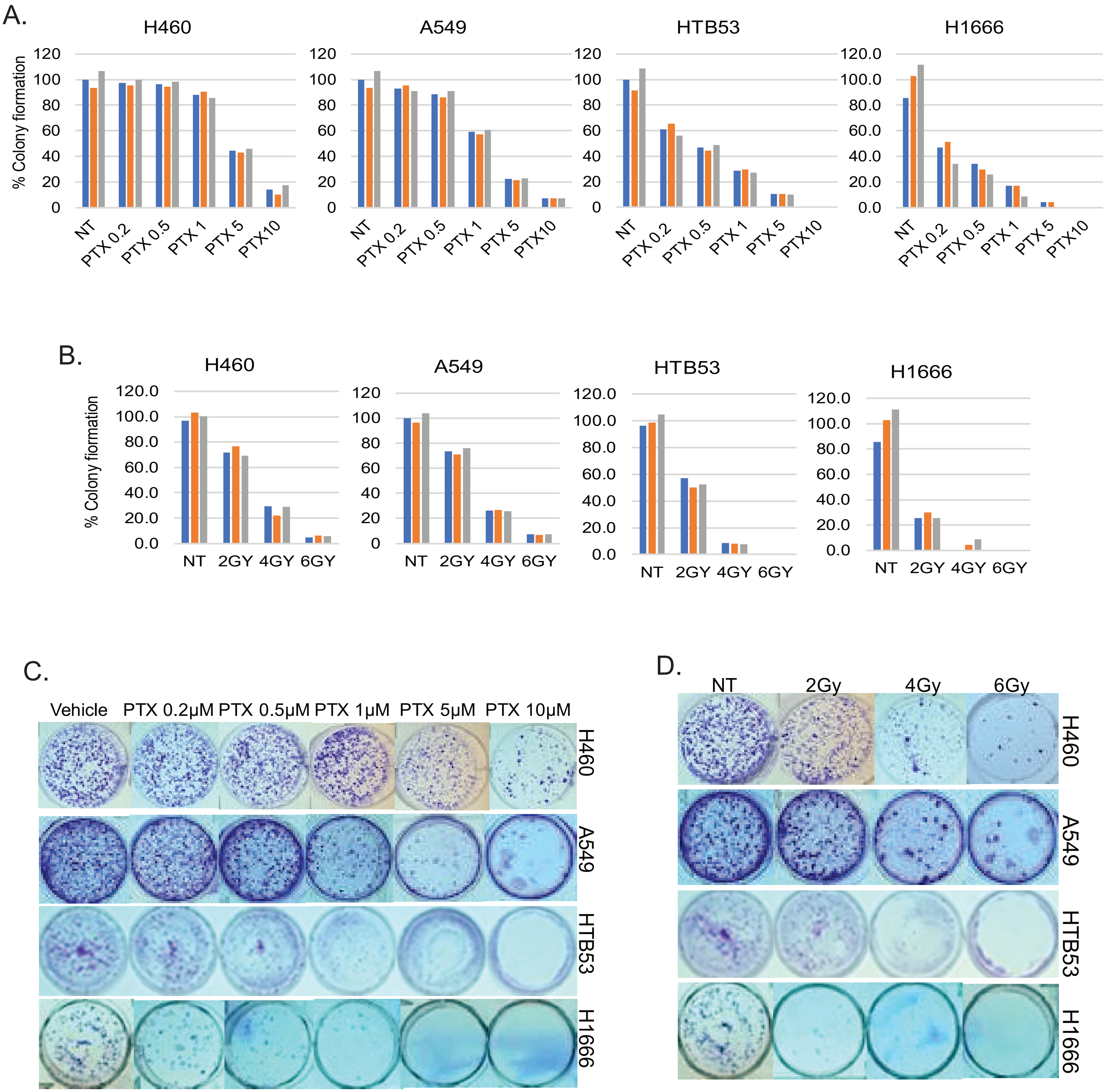
A.
H460
A549
HTB53
H1666
% Colony fiormation
NT
PTX 0.2
PTX 0.5
PTX 1
PTX 5
PTX10
B.
NT
2GY
4GY
6GY
C.
Vehicle
PTX 0.2μM
PTX 0.5μM
PTX 1μM
PTX 5μM
PTX 10μM
D.
2Gy
4Gy
6Gy

Figure S3

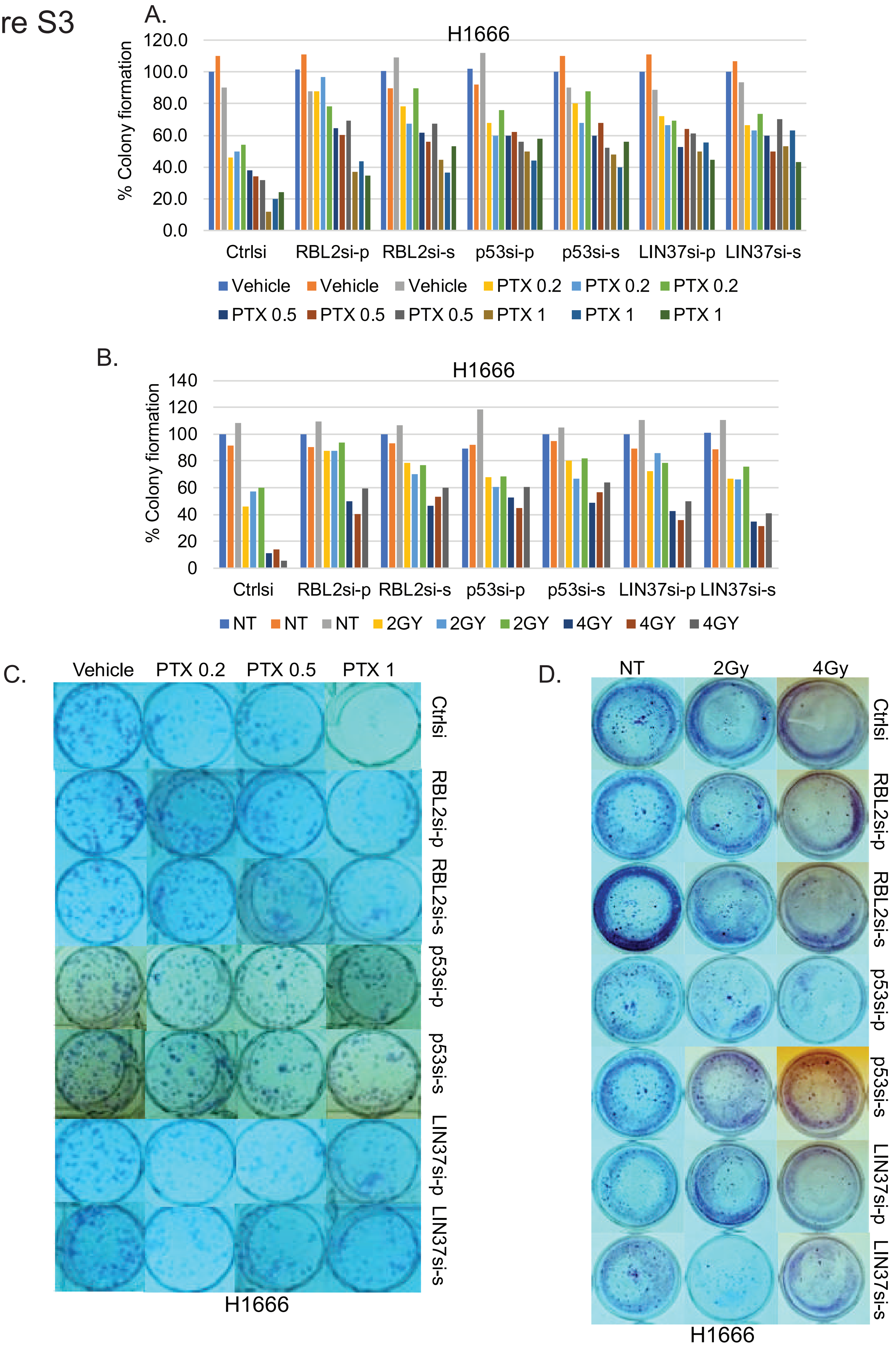

A.
H1666
% Colony fiormation
120.0
100.0
80.0
60.0
40.0
20.0
0.0
Ctrlsi
RBL2si-p
RBL2si-s
p53si-p
p53si-s
LIN37si-p
LIN37si-s
Vehicle
Vehicle
Vehicle
PTX 0.2
PTX 0.2
PTX 0.2
PTX 0.5
PTX 0.5
PTX 0.5
PTX 1
PTX 1
PTX 1
B.
H1666
% Colony fiormation
140
120
100
80
60
40
20
0
Ctrlsi
RBL2si-p
RBL2si-s
p53si-p
p53si-s
LIN37si-p
LIN37si-s
NT
NT
NT
2GY
2GY
2GY
4GY
4GY
4GY
C.
Vehicle
PTX 0.2
PTX 0.5
PTX 1
Ctrlsi
RBL2si-p
RBL2si-s
p53si-p
p53si-s
LIN37si-p
LIN37si-s
H1666
D.
NT
2Gy
4Gy
Ctrlsi
RBL2si-p
RBL2si-s
p53si-p
p53si-s
LIN37si-p
LIN37si-s
H1666

Figure S4

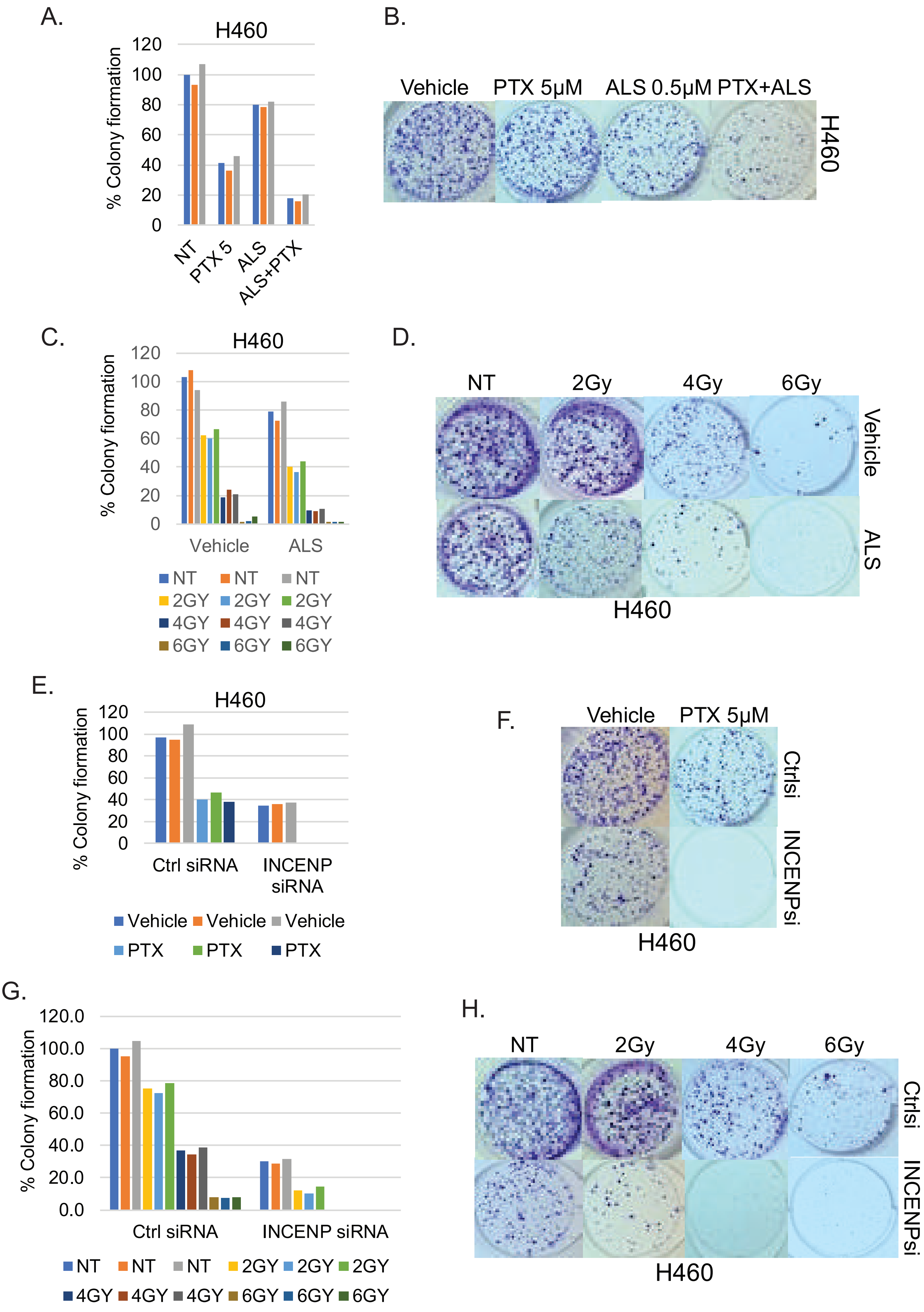

A.
H460
% Colony fiormation
120
100
80
60
40
20
0
NT
PTX 5
ALS
ALS+PTX
B.
Vehicle
PTX 5μM
ALS 0.5μM
PTX+ALS
H460
C.
H460
% Colony fiormation
Vehicle
ALS
NT NT NT
2GY 2GY 2GY
4GY 4GY 4GY
6GY 6GY 6GY
D.
NT
2Gy
4Gy
6Gy
Vehicle
ALS
H460
E.
H460
% Colony fiormation
Ctrl siRNA
INCENP siRNA
Vehicle Vehicle Vehicle
PTX PTX PTX
F.
Vehicle
PTX 5μM
Ctrlsi
INCENPsi
H460
G.
% Colony fiormation
120.0
100.0
80.0
60.0
40.0
20.0
0.0
Ctrl siRNA
INCENP siRNA
NT NT NT 2GY 2GY 2GY
4GY 4GY 4GY 6GY 6GY 6GY
H.
NT
2Gy
4Gy
6Gy
Ctrlsi
INCENPsi
H460

Figure S5

Orinigal filrs for FIgure 5D

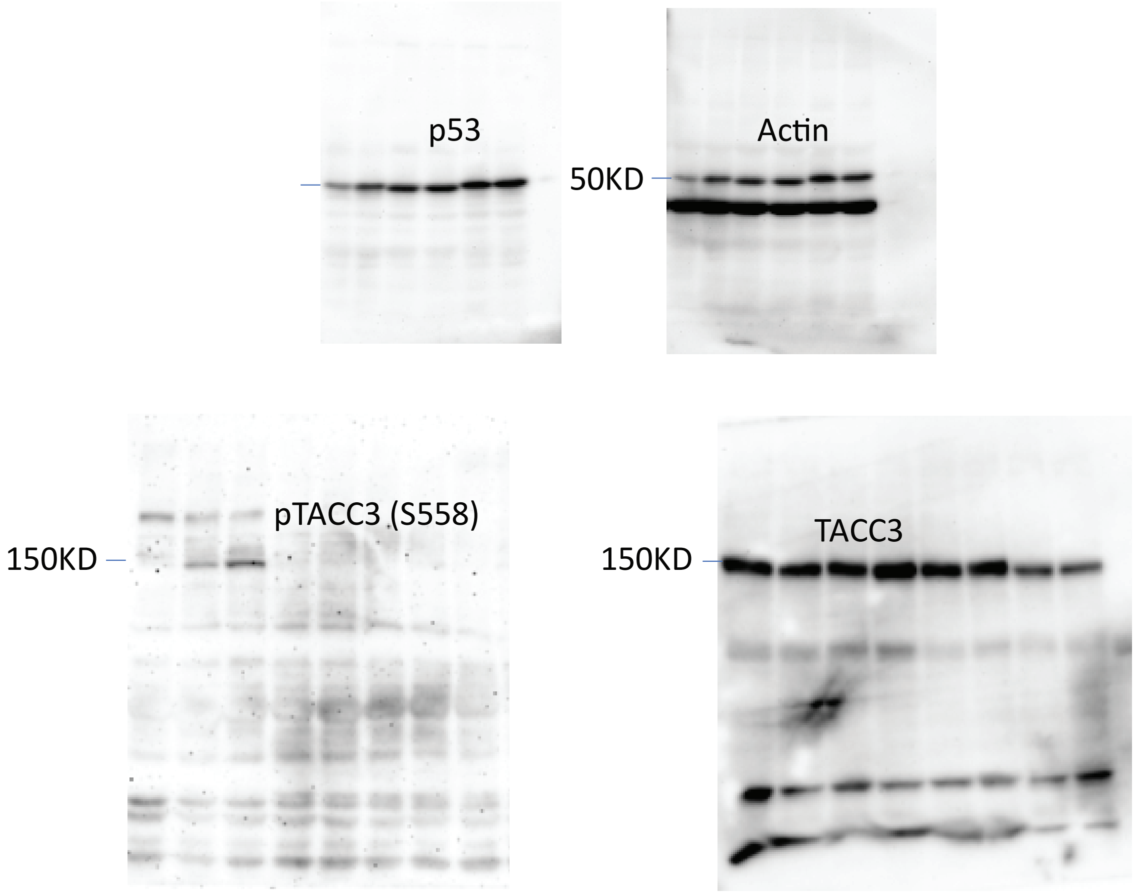

Orinigal filrs for FIgure 5G

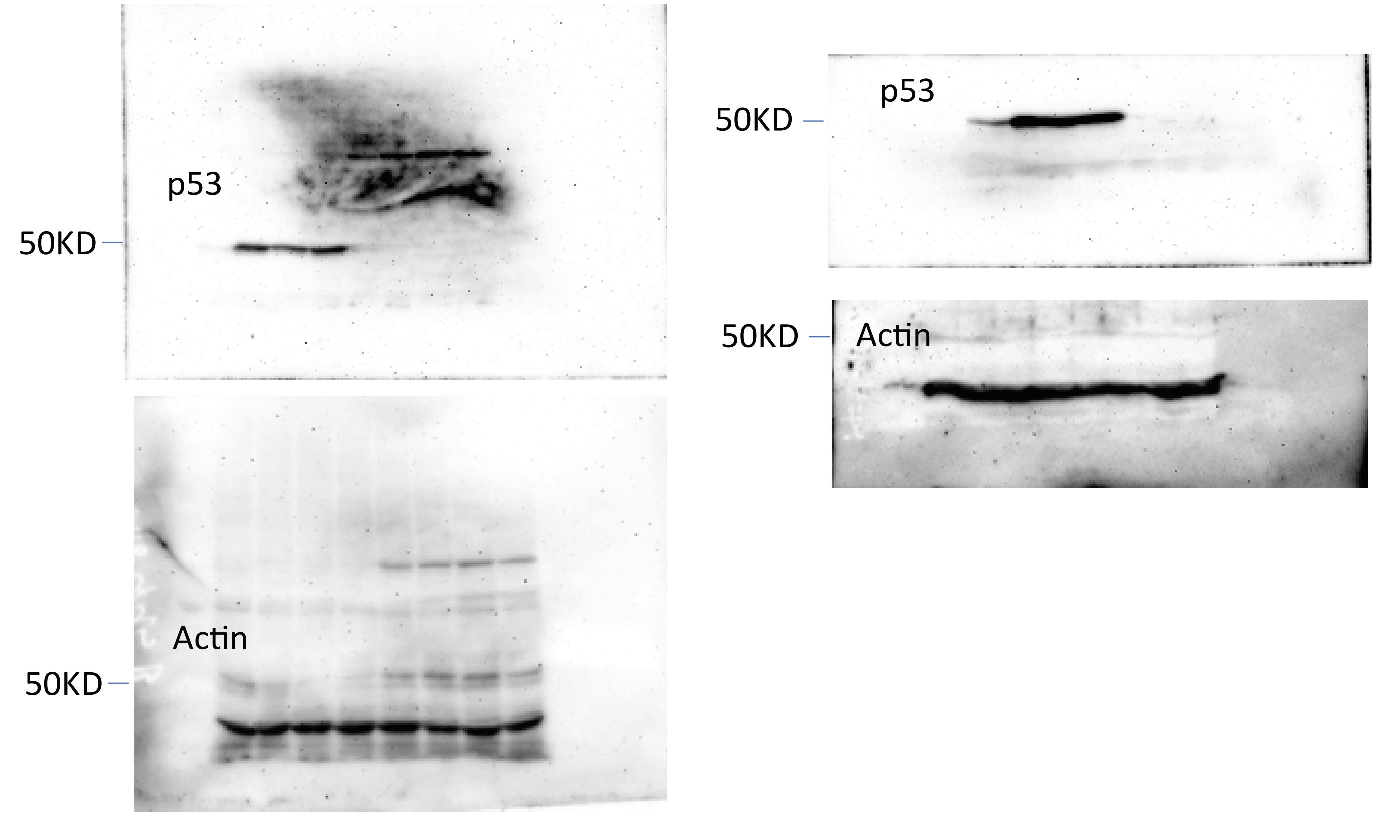

Figure S6

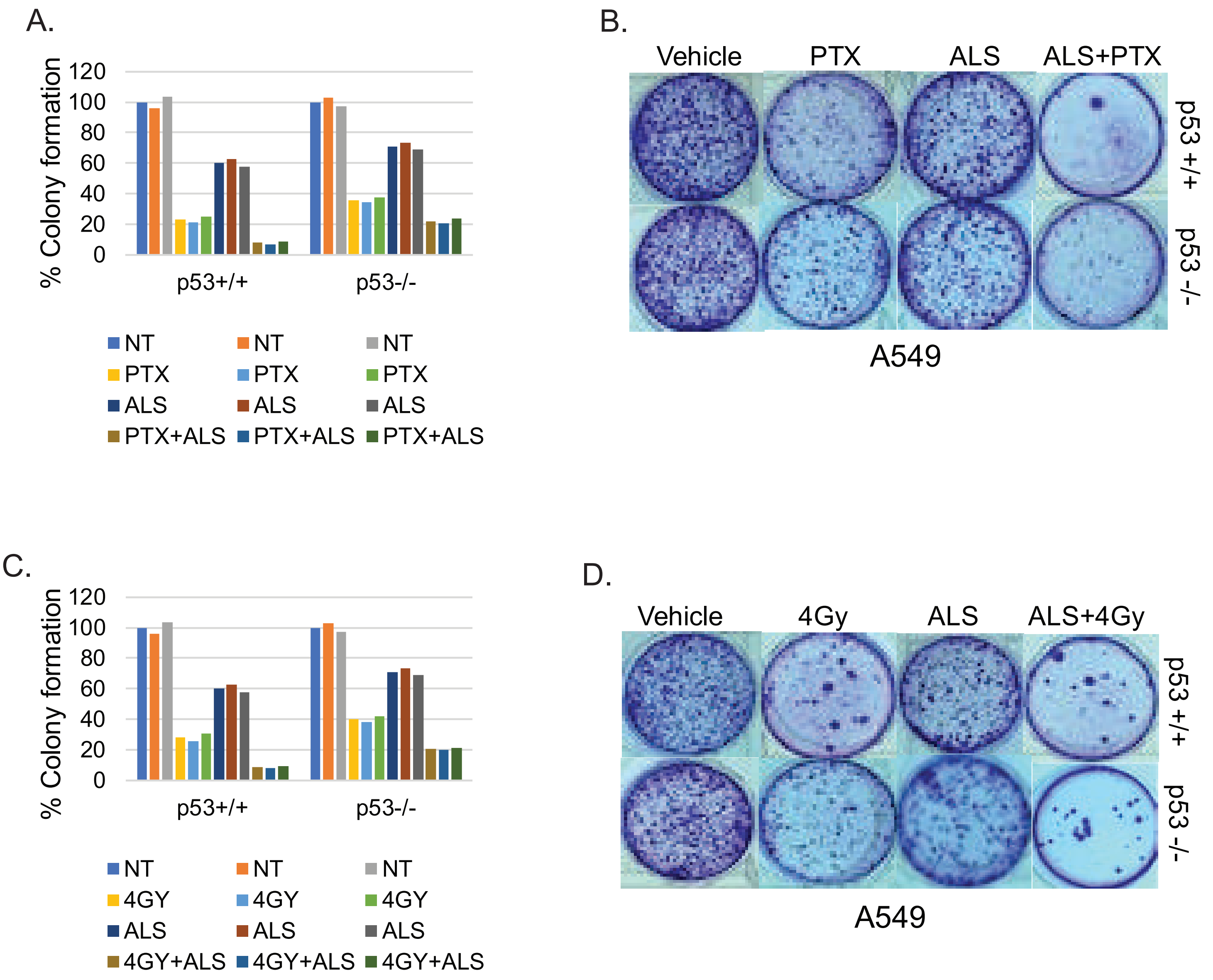
A.
% Colony formation
120
100
80
60
40
20
0
p53+/+
p53-/-
NT
NT
NT
PTX
PTX
PTX
ALS
ALS
ALS
PTX+ALS
PTX+ALS
PTX+ALS
B.
Vehicle
PTX
ALS
ALS+PTX
p53 +/+
p53 -/-
A549
C.
% Colony formation
120
100
80
60
40
20
0
p53+/+
p53-/-
NT
NT
NT
4GY
4GY
4GY
ALS
ALS
ALS
4GY+ALS
4GY+ALS
4GY+ALS
D.
Vehicle
4Gy
ALS
ALS+4Gy
p53 +/+
p53 -/-
A549

Table S1 : Genes negatively correlated with RBL2 in WT p53 tumors

	WT p53 NSCLC	Mut p53 NSCLC		WT p53 NSCLC	Mut p53 NSCLC
Gene Names	Pearson with RBL2	Pearson with RBL2	Gene Names	Pearson with RBL2	Pearson with RBL2
MKI67	-0.20	-0.04	CENPU	-0.28	-0.15
CENPE	-0.22	-0.05	DEPDC1	-0.26	-0.15
C5orf34	-0.22	-0.05	EXO1	-0.24	-0.15
CENPI	-0.2	-0.06	FOXM1	-0.23	-0.15
CENPN	-0.22	-0.07	SPAG5	-0.26	-0.16
INCENP	-0.20	-0.07	CDC45	-0.27	-0.16
FAM72A	-0.23	-0.08	CEP55	-0.28	-0.16
IQGAP3	-0.28	-0.08	MND1	-0.24	-0.16
KIF14	-0.21	-0.08	RAD51AP1	-0.2	-0.16
PLK4	-0.21	-0.08	CENPL	-0.24	-0.17
CENPF	-0.21	-0.09	CENPH	-0.24	-0.17
CDCA8	-0.27	-0.1	GGH	-0.25	-0.17
KIF11	-0.23	-0.11	MCM4	-0.2	-0.17
ARHGEF39	-0.23	-0.11	PRR11	-0.27	-0.17
FAM72B	-0.26	-0.11	UNG	-0.2	-0.17
ARHGA11A	-0.2	-0.12	CDCA2	-0.22	-0.18
DIAPH3	-0.22	-0.12	CDT1	-0.24	-0.18
HMMR	-0.25	-0.12	CHEK1	-0.24	-0.18
NEIL3	-0.23	-0.12	FAM72D	-0.31	-0.18
STIL	-0.2	-0.12	GTSE1	-0.27	-0.18
ANLN	-0.27	-0.13	MELK	-0.23	-0.18
ESCO2	-0.2	-0.14	PARPBP	-0.22	-0.18
ESPL1	-0.25	-0.14	TTK	-0.24	-0.18
CIP2A	-0.2	-0.14	ZNF714	-0.23	-0.18
KIF15	-0.2	-0.14	BUB1B	-0.24	-0.19
RAD54B	-0.22	-0.14	KIF4A	-0.28	-0.19
RAD54L	-0.23	-0.14	CDC25C	-0.33	-0.19
TONSL	-0.2	-0.14	NUF2	-0.28	-0.19
ERCC6L	-0.27	-0.15	PRC1	-0.25	-0.19

Table S2 : Genes negatively correlated with RBL2 in WT p53 tumors and in mutant p53 tumors

	WT p53 NSCLC	Mut p53 NSCLC		WT p53 NSCLC	Mut p53 NSCLC		WT p53 NSCLC	Mut p53 NSCLC
Gene Names	Pearson with RBL2	Pearson with RBL2	Gene Names	Pearson with RBL2	Pearson with RBL2	Gene Names	Pearson with RBL2	Pearson with RBL2
KIF23	-0.24	-0.20	KCNK5	-0.31	-0.22	TROAP	-0.31	-0.25
NUSAP1	-0.23	-0.20	MIS18BP1	-0.23	-0.22	CENPM	-0.3	-0.26
GINS1	-0.24	-0.2	ORC1	-0.25	-0.22	CEP295	-0.29	-0.26
KIF18B	-0.24	-0.2	PBK	-0.26	-0.22	DLGAP5	-0.3	-0.26
MIS18A	-0.24	-0.2	PKMYT1	-0.32	-0.22	H2AFZ	-0.33	-0.26
NCAPG	-0.24	-0.2	PSRC1	-0.26	-0.22	HIURP	-0.29	-0.26
NDC80	-0.3	-0.2	TRAIP	-0.27	-0.22	KIF2C	-0.33	-0.26
NEK2	-0.26	-0.2	ZNF92	-0.31	-0.22	OIP5	-0.26	-0.26
SUV39H1	-0.28	-0.2	ZWINT	-0.29	-0.22	UBE2T	-0.32	-0.26
TRIP13	-0.26	-0.2	AUNIP	-0.28	-0.23	SPC24	-0.28	-0.27
NCAPH	-0.23	-0.21	CDC25A	-0.24	-0.23	AURKB	-0.29	-0.28
PLK1	-0.29	-0.21	CDK1	-0.27	-0.23	CENPW	-0.33	-0.28
SGOL1	-0.28	-0.21	CIT	-0.23	-0.23	FEN1	-0.26	-0.28
TPX2	-0.28	-0.21	KIF20A	-0.31	-0.23	LSM5	-0.31	-0.28
ASF1B	-0.28	-0.21	KIFC1	-0.27	-0.23	PPIH	-0.24	-0.28
BUB1	-0.21	-0.21	H2AFX	-0.34	-0.24	TKI	-0.32	-0.28
TEDC1	-0.29	-0.21	MDA2L1	-0.28	-0.24	CCNB2	-0.3	-0.29
CCNA2	-0.28	-0.21	SKA3	-0.34	-0.24	CENPA	-0.29	-0.29
E2F1	-0.22	-0.21	CDCA5	-0.27	-0.25	MYBL2	-0.24	-0.29
LRR1	-0.27	-0.21	DSCC1	-0.21	-0.25	RNASEH2A	-0.28	-0.3
MCM10	-0.24	-0.21	KNSTRN	-0.25	-0.25	SPC25	-0.28	-0.3
RECQL4	-0.26	-0.21	MTFR2	-0.27	-0.25	UBE2C	-0.34	-0.3
SGO1	-0.28	-0.21	PIMREG	-0.27	-0.25	BIRC5	-0.32	-0.31
SKA1	-0.28	-0.21	POC1A	-0.36	-0.25	CDC20	-0.38	-0.33
AURKA	-0.24	-0.22	RAPGAP1	-0.24	-0.25	CKS2	-0.36	-0.33
TEDC2	-0.33	-0.22	RAD51	-0.29	-0.25	NUDT1	-0.33	-0.33
CDCA3	-0.27	-0.22	SLC25A10	-0.38	-0.25	DTYMK	-0.43	-0.37
DEPDC1B	-0.24	-0.22						

Table S3. Expression levels of AURKA/B pathway genes in WT p53 vs. mutant p53 NSCLC

	WT p53 NSCLC	Mut p53 NSCLC	
Gene Names	Median (FKPM) ± STDEV	Median (FKPM) ± STDEV	p Value
**ANLN**	3.7 ± 5.7	11.2 ± 9.2	2.25E-33
**AURKA**	5.3 ± 6.2	11 ± 8.1	1.84E-21
**AURKB**	3.7 ± 6.9	10.5 ± 8.3	8.69E-29
**BUB1B**	2.1 ± 3.3	6.3 ± 4.2	1.32E-36
**CDC20**	9.8 ± 17	28.9 ± 20	6.08E-35
**CENPE**	0.8 ± 1	1.9 ± 1.4	1.38E-32
**CENPF**	2.5 ± 4	6.9 ± 5.6	1.57E-30
**CENPL**	1.4 ± 1	2.5 ± 1.5	5.64E-29
**CENPN**	2.2 ± 2.6	4.8 ± 3.4	4.56E-30
**ERCC6L**	0.7 ± 0.9	1.8 ± 1.6	3.05E-25
**INCENP**	4.2 ± 3.4	7.9 ± 4.5	3.05E-32
**KIF11**	3.6 ± 4.7	8.8 ± 5.1	3.65E-35
**KIF23**	1.7 ± 2.8	5.7 ± 3.4	2.41E-44
**KIF4A**	2.5 ± 3.6	7.2 ± 4.2	1.46E-42
**MKI67**	3.6 ± 5.8	8.7 ± 6.3	1.38E-23
**NCAPH**	2.5 ± 3.9	8.1 ± 5.3	7.61E-43
**NUSAP1**	7.4 ± 9.2	17.5 ± 12	1.09E-33
**PLK1**	3.7 ± 5.2	9.3 ± 5.7	2.59E-31
**SPAG5**	3.6 ± 4.2	8.1 ± 5.9	1.83E-29
**SGOL1**	0.7 ± 1	1.8 ± 1.1	1.57E-30
**TACC3**	5.9 ± 5.3	9.7 ± 7.8	1.49E-14
**TPX2**	9.2 ± 15.2	28.8 ± 23	2.28E-35

Table S4. Positive correlation of AURKA with other genes in WT p53 vs. mutant p53 NSCLC

	WT p53 NSCLC	Mut p53 NSCLC
Gene Names	Pearson with AURKA	Pearson with AURKA
**ANLN**	0.68	0.35
**AURKB**	0.71	0.49
**BUB1B**	0.73	0.5
**CDC20**	0.74	0.53
**CENPE**	0.71	0.45
**CENPF**	0.67	0.42
**CENPL**	0.62	0.36
**CENPN**	0.63	0.38
**ERCC6L**	0.75	0.34
**INCENP**	0.67	0.41
**KIF11**	0.69	0.46
**KIF23**	0.73	0.49
**KIF4A**	0.77	0.56
**MKI67**	0.66	0.38
**NCAPH**	0.76	0.52
**NUSAP1**	0.72	0.49
**PLK1**	0.73	0.53
**SPAG5**	0.77	0.56
**SGOL1**	0.78	0.55
**TACC3**	0.61	0.32
**TPX2**	0.76	0.44

Table S5	A	B	C	t-test (A and B)	t-test (A and C)
**H1666**	**Ctrl siRNA**	**p53 siRNA-p**	**p53 siRNA-s**	**p values**	**p values**
**AURKA**	1	1.90	1.65	0.001	0.004
**AURKB**	1	1.98	1.68	0.002	0.002
**CENPE**	1	2.17	1.54	0.001	0.019
**CENPF**	1	1.71	1.78	0.002	0.006
**CENPN**	1	1.99	1.65	0.002	0.004
**ERCC6L**	1	2.09	1.79	0.001	0.003
**INCENP**	1	1.95	1.66	0.001	0.012
**KIF4A**	1	1.98	1.67	0.000	0.008
**KIF23**	1	1.98	1.66	0.000	0.010
**MKI67**	1	1.70	1.57	0.004	0.007
**NCAPH**	1	1.88	1.61	0.006	0.009
**NUSAP1**	1	2.20	1.87	0.002	0.002
**PLK1**	1	2.17	1.73	0.005	0.001
**SGOL1**	1	1.79	1.61	0.006	0.003
**SPAG5**	1	1.94	1.64	0.007	0.009
**TACC3**	1	1.63	1.42	0.005	0.049

Table S6	A	B	C	t-test (A and B)	t-test (A and C)
**H1666**	**Ctrl siRNA**	**RBL2 siRNA-p**	**RBL2 siRNA-s**	**p values**	**p values**
**AURKA**	1	1.80	1.72	0.001	0.001
**AURKB**	1	1.51	1.37	0.003	0.004
**CENPE**	1	1.87	1.70	0.001	0.005
**CENPF**	1	1.48	1.31	0.007	0.004
**CENPN**	1	1.39	1.37	0.014	0.012
**ERCC6L**	1	1.69	1.56	0.003	0.006
**INCENP**	1	1.61	1.42	0.004	0.007
**KIF4A**	1	1.61	1.77	0.007	0.007
**KIF23**	1	1.72	1.65	0.001	0.003
**MKI67**	1	1.54	1.61	0.007	0.011
**NCAPH**	1	1.53	1.42	0.017	0.006
**NUSAP1**	1	1.49	1.82	0.038	0.012
**PLK1**	1	1.51	1.89	0.004	0.010
**SGOL1**	1	1.69	1.57	0.008	0.004
**SPAG5**	1	1.74	1.69	0.011	0.004
**TACC3**	1	1.43	1.31	0.011	0.071

Table S7	A	B	C	t-test (A and B)	t-test (A and C)
**H1666**	**Ctrl siRNA**	**LIN37 siRNA-p**	**LIN37 siRNA-s**	**p values**	**p values**
**AURKA**	1	1.61	1.43	0.003	0.010
**AURKB**	1	1.87	1.63	0.001	0.008
**CENPE**	1	1.77	1.48	0.006	0.008
**CENPF**	1	1.45	1.78	0.014	0.002
**CENPN**	1	1.59	1.65	0.007	0.002
**ERCC6L**	1	1.88	1.66	0.001	0.009
**INCENP**	1	1.58	1.35	0.014	0.024
**KIF4A**	1	2.04	1.91	0.001	0.001
**KIF23**	1	1.85	1.87	0.003	0.011
**MKI67**	1	1.71	1.91	0.010	0.006
**NCAPH**	1	2.19	2.12	0.001	0.002
**NUSAP1**	1	2.11	1.87	0.002	0.004
**PLK1**	1	1.99	1.73	0.004	0.007
**SGOL1**	1	1.47	1.66	0.003	0.006
**SPAG5**	1	1.84	1.62	0.007	0.005
**TACC3**	1	1.61	1.36	0.012	0.025

Table S8	A	B	C	t-test (A and B)	t-test (A and C)
**H2030**	**Ctrl siRNA**	**RBL2 siRNA-p**	**RBL2 siRNA-s**	**p values**	**p values**
**AURKA**	1	1.40	1.24	0.050	0.001
**AURKB**	1	0.69	0.53	0.058	0.004
**CENPE**	1	0.87	0.78	0.335	0.005
**CENPF**	1	0.59	0.76	0.027	0.004
**CENPN**	1	0.71	0.64	0.001	0.012
**ERCC6L**	1	0.75	0.67	0.293	0.006
**INCENP**	1	0.61	0.51	0.000	0.007
**KIF4A**	1	0.57	0.53	0.014	0.007
**KIF23**	1	0.54	0.55	0.057	0.003
**MKI67**	1	0.95	0.66	0.562	0.011
**NCAPH**	1	1.56	1.27	0.006	0.006
**NUSAP1**	1	1.53	1.09	0.115	0.012
**PLK1**	1	1.66	1.30	0.001	0.010
**SGOL1**	1	1.45	1.33	0.007	0.004
**SPAG5**	1	1.27	1.15	0.185	0.004
**TACC3**	1	1.04	0.98	0.659	0.712

Table S9	A	B	C	t-test (A and B)	t-test (A and C)
**H2030**	**Ctrl siRNA**	**LIN37 siRNA-p**	**LIN37 siRNA-s**	**p values**	**p values**
**AURKA**	1	1.13	1.29	0.061	0.035
**AURKB**	1	1.27	0.85	0.000	0.089
**CENPE**	1	0.38	0.66	0.002	0.002
**CENPF**	1	1.03	1.26	0.790	0.054
**CENPN**	1	0.54	0.85	0.001	0.028
**ERCC6L**	1	1.50	1.17	0.091	0.017
**INCENP**	1	0.63	0.94	0.004	0.313
**KIF4A**	1	0.32	0.53	0.000	0.001
**KIF23**	1	0.24	0.53	0.000	0.002
**MKI67**	1	0.57	0.78	0.011	0.014
**NCAPH**	1	0.36	0.55	0.001	0.001
**NUSAP1**	1	0.31	0.46	0.000	0.000
**PLK1**	1	0.29	0.55	0.000	0.001
**SGOL1**	1	1.31	1.39	0.011	0.026
**SPAG5**	1	0.48	0.85	0.000	0.042
**TACC3**	1	0.83	0.71	0.181	0.001

Table S10	A	B	C	t-test (A and B)	t-test (A and C)
**H460**	**Ctrl siRNA**	**p53 siRNA-p**	**p53 siRNA-s**	**p values**	**p values**
**AURKA**	1	1.58	1.34	0.003	0.028
**AURKB**	1	1.49	1.30	0.043	0.012
**CENPE**	1	1.69	1.34	0.007	0.022
**CENPF**	1	1.32	1.23	0.024	0.038
**CENPN**	1	1.45	1.30	0.011	0.061
**ERCC6L**	1	2.13	1.83	0.011	0.001
**INCENP**	1	1.43	1.22	0.021	0.045
**KIF4A**	1	1.57	1.47	0.003	0.003
**KIF23**	1	1.42	1.44	0.007	0.024
**MKI67**	1	1.00	1.03	0.965	0.094
**NCAPH**	1	1.37	1.33	0.005	0.010
**NUSAP1**	1	1.55	1.32	0.005	0.041
**PLK1**	1	1.49	1.32	0.003	0.023
**SGOL1**	1	1.55	1.54	0.012	0.014
**SPAG5**	1	1.55	1.40	0.007	0.023
**TACC3**	1	1.24	1.31	0.046	0.016

Table S11	A	B	C	t-test (A and B)	t-test (A and C)
**H460**	**Ctrl siRNA**	**RBL2 siRNA-p**	**RBL2 siRNA-s**	**p values**	**p values**
**AURKA**	1	1.30	1.22	0.006	0.011
**AURKB**	1	1.11	1.20	0.060	0.028
**CENPE**	1	1.47	1.30	0.004	0.026
**CENPF**	1	1.11	1.18	0.060	0.096
**CENPN**	1	1.19	1.17	0.057	0.051
**ERCC6L**	1	1.45	1.32	0.008	0.018
**INCENP**	1	1.32	1.33	0.010	0.005
**KIF4A**	1	1.20	1.22	0.022	0.024
**KIF23**	1	1.12	1.13	0.029	0.085
**MKI67**	1	0.94	1.01	0.346	0.856
**NCAPH**	1	1.03	0.92	0.714	0.137
**NUSAP1**	1	1.34	1.25	0.020	0.002
**PLK1**	1	1.15	1.18	0.220	0.068
**SGOL1**	1	1.29	1.17	0.044	0.039
**SPAG5**	1	1.34	1.29	0.050	0.021
**TACC3**	1	1.13	1.19	0.105	0.028

Table S12	A	B	t-test (A and B)
A549	p53+/+	p53-/-	p values
AURKA	1	1.49	0.041
AURKB	1	1.66	0.034
CENPE	1	2.17	0.012
CENPF	1	1.21	0.066
CENPN	1	1.33	0.024
ERCC6L	1	2.33	0.007
INCENP	1	1.35	0.123
KIF4A	1	1.76	0.007
KIF23	1	1.77	0.010
MKI67	1	1.43	0.067
NCAPH	1	1.50	0.024
NUSAP1	1	1.44	0.024
PLK1	1	1.76	0.007
SGOL1	1	1.41	0.007
SPAG5	1	1.53	0.009
TACC3	1	1.19	0.042

Table S13	2GY	4GY	PTX 0.2	PTX 0.5	PTX 1
Fig. 6A and B	p values	p Values	p Values	p Values	p Values
Ctrl siRNA and RBL2 siRNA-p	0.032	0.038	0.031	0.014	0.050
Ctrl siRNA and RBL2 siRNA-s	0.011	0.018	0.038	0.023	0.034
Ctrl siRNA and p53 siRNA-p	0.053	0.027	0.046	0.005	0.017
Ctrl siRNA and p53 siRNA-s	0.048	0.019	0.033	0.029	0.026
Ctrl siRNA and LIN37 siRNA-p	0.016	0.039	0.029	0.038	0.029
Ctrl siRNA and LIN37 siRNA-s	0.047	0.041	0.016	0.061	0.046

Table S14	t test	p values
Fig.5E	**NT and PTX**	0.0005
Fig.5E	**NT and ALS**	0.0204
Fig.5E	**PTX and ALS+PTX**	0.0033
Fig.5E	**ALS and ALS+PTX**	0.0000
Fig.5E	**2GY and ALS+2GY**	0.0005
Fig.5E	**4GY and ALS+4GY**	0.0238
Fig.5E	**6GY and ALS+6GY**	0.2923

	Table S15	Vehicle	PTX	2GY	4GY	6GY
	t test	p Value	p Value	p Value	p Value	p Value
Fig.5F	**Ctrl siRNA and INCENP siRNA**	0.003582	0.003557	7.45408E-05	0.001096	0.000493

Table S16	t test	p values
Fig. 5H	**p53+/+ NT and PTX**	0.000
Fig. 5H	**p53+/+ NT and ALS**	0.008
Fig. 5H	**p53+/+ PTX and PTx+ALS**	0.002
Fig. 5H	**p53+/+ ALS and PTX+ALS**	0.001
Fig. 5H	**p53-/- NT and PTX**	0.002
Fig. 5H	**p53-/- NT and ALS**	0.000
Fig. 5H	**p53-/- PTX and PTx+ALS**	0.000
Fig. 5H	**p53-/- ALS and PTX+ALS**	0.002
Fig. 5H	**p53+/+ PTX and p53-/- PTX**	0.001
Fig. 5H	**p53+/+ ALS and p53-/- ALS**	0.000
Fig. 5H	**p53+/+ PTX+ALS and p53-/- PTX+ALS**	0.001
Fig. 5H	**p53+/+ NT and 4GY**	8E-05
Fig. 5H	**p53+/+ 4GY and ALS+4GY**	0.003
Fig. 5H	**p53+/+ ALS and ALS+4GY**	0.001
Fig. 5H	**p53-/- NT and 4GY**	0.002
Fig. 5H	**p53-/- 4GY and ALS+4GY**	0.001
Fig. 5H	**p53-/- ALS and ALS+ 4GY**	0.001
Fig. 5H	**p53+/+ 4GY and p53-/- 4GY**	0.001
Fig. 5H	**p53+/+ 4GY+ALS and p53-/- 4GY+ALS**	0.000
